# Assessing Knowledge, Attitude and Practices for Oral Squamous Cell Carcinoma among Health Care Professionals in Princess Nourah University, Riyadh, KSA

**DOI:** 10.31557/APJCP.2020.21.2.539

**Published:** 2020

**Authors:** Farhat Kazmi, Shahad Alkait, Hend Alghamdi, Ghaida Alhussain, Afsheen Tabassum

**Affiliations:** 1 *Department of Oral Diagnosis, College of Dentistry, Princess Nourah Bint Abdulrahman University, Riyadh, *; 2 *Department of Preventive Dental Sciences, College of Dentistry, Imam Abdulrahman Bin Faisal University, Dammam, KSA. *

**Keywords:** Awareness, knowledge, attitude and practices, early detection, oral cancer

## Abstract

**Background:**

Oral Squamous Cell Carcinoma (OSCC) is a growing public health problem affecting 2.2 million of the world’s population per year and the rates are increasing annually. The disease is usually diagnosed in later stages, and carries high morbidity and mortality rates worldwide. Proper awareness among health care professionals (HCP) is the most significant factor for ensuring early diagnosis and treatment. They should have thorough knowledge to identify all suspicious lesions or otherwise to seek specialist opinion when unsure. The present study aimed to assess the knowledge, attitude, and practices (KAPs) of OSCC among dental and medical undergraduate students along with general practitioners and specialists of both disciplines.

**Materials and Methods:**

The present cross-sectional study was conducted at Princess Nourah Bint Abdulrahman University and its affiliated hospital. A total of 332 participants filled a close-ended online questionnaire. Responses to the questionnaire were analyzed using descriptive and analytical statistics.

**Results:**

Of the 450 health care professionals approached, 332 filled the questionnaire with a response rate of 73.77.%. It was observed that the mean knowledge index was higher among dental participants (10.96 ± 1.85). The attitude index was higher at medical side (6.89 ± 1.11), and the practice index was also higher among the dental participants (4.95 ± 0.91). Most of the health care professionals had knowledge regarding risk factors associated with OSCC. HCPs indicated their lack of training as the main barrier for conducting a comprehensive examination for OSCC. Interestingly, the vast majority of HCPs expressed their interest to have further educational and training sessions regarding this malignancy.

**Conclusion:**

The study puts forward, the need for intensive training and workshops for awareness and improvement of the abilities of the HCPs, (including dental and medical undergraduate students along with practitioners and specialists) to diagnose OSCC.

## Introduction

Oral cancer (OC) is one of the most common cancers that dominates globally and is known for its lethal effects and disastrous outcomes (Jin et al., 2016). Oral Squamous cell carcinoma (OSCC) constitutes about ninety percent of all oral malignancies (Feller et al., 2013) and is ranked as the eighth most common cancer world-wide (Abdulla et al., 2018). The incidence of OSCC is rapidly increasing worldwide (Bray et al., 2018) with higher incidence and mortality rate in developing countries as compared to developed nations (Ferlay et al., 2010). It is one of the most common types of cancers in South Asian countries like India, Sri Lanka, Pakistan, and Bangladesh and contributes to nearly one-fourth of all new cases (Gupta et al., 2016). 

In recent years, there has been a significant increase in the OSCC in Saudi Arabia also; it has been estimated that it is the 3rd most common cause of malignancy after lymphoma and leukemia in Saudia Arabia (Eltelety et al., 2014). The age of onset and the site involved varies among different regions. Usually OSCC is seen at the age of 40 or above however surprisingly since last decade, an increase in the percentage of young patients having OSCC has been reported in literature (Ghantous and Abu Elnaaj, 2017, Silverman et al., 2010). The buccal (cheek) mucosa has been found to be the most common site for OSCC in South and Southeast Asia; whereas, in all other regions, tongue is the most common site reported (Forman et al., 2013). The common risk factors for the development of OSCC include tobacco smoking/chewing, betel-quid chewing with or without tobacco, and alcohol use (Sankaranarayanan et al., 2015, Hashibe et al., 2007). Regional variations in incidence and the site of occurrence are correlated with causes like alcohol and smoking in Western countries, while betel quid and tobacco chewing is the most common predisposing factor in South and Southeast Asia (Lambert et al., 2011). 

OSCC may develop from premalignant lesions/conditions of oral cavity and sometimes it develops de-novo. In initial stages, the lesions are usually painless and do not produce any discomfort and thus are frequently undiagnosed till they become symptomatic (Neville and Day, 2002). In spite of the latest innovations and advanced technologies regarding diagnosis and treatment, OSCC still continues to be a major health problem in many parts of the world (Forman et al., 2013). High mortality and morbidity rates in OSCC is due to the fact that they are diagnosed as late stage, mostly when invasion and metastasis has already occurred.

OSCC patients are initially examined by Health care professionals (HCP) and early diagnosis highly depends on their knowledge. Early detection and prevention of the disease correlates with better prognosis and improves the survival rates (Van der Waal, 2013; Friedrich, 2010). Therefore, it is vital that all HCP should have good basic knowledge of OSCC. Hence, the aim of the study was to investigate the knowledge of dental students, medical students, and practitioners (of medical and dental background) about OSCC and to assess their attitude and practices for managing oral cancer.

## Materials and Methods

The present cross-sectional study was conducted to assess the knowledge, attitude and practice of OSCC among dental and medical students, dental and medical general practitioners (GPs), and specialists working at Princess Nourah Bint Abdulrahman University and its affiliated hospital. Ethical approval to carry out the study was obtained from the Institutional Review Board of the College of dentistry, Princess Nourah University (IRB number:180196). 

The sample size was calculated by using the Research Advisors-2006; with a confidence interval of 95%, a margin of error of 5% and an estimated proportion of the population (p=0.25). By using Cochrane’s sample size formula, the calculated minimal sample size was 285. To allow for attrition and missing data, we inflated the sample size by 10%.The study was carried out over a period of 6 months. Informed consent was taken from the participants prior to the study. The participation in study was voluntary and confidentiality of the responses was assured to participants. 

We developed an online google survey form with little modifications in previously used questionnaire (Jnaneswar et al., 2017). The link to the questionnaire was sent to all students and practitioners via private SMS, and e-mail according to the last cell numbers and email addresses available in the HR and academic database. A reminder was sent every week for a total of three reminders per participant. The questionnaire was divided into various sections including socio-demographic data, their knowledge, attitude and practices towards OSCC. The socio-demographic data section in the questionnaire included participants’ demographic details, educational background, years of experience, the year of study (for students), number of patients encountered each week and number of dental referrals for oral problems. KAP section included 15 questions regarding OSCC knowledge, seven questions related to attitude and six questions related to practices regarding this malignancy. The questionnaire was reviewed by two experts to ensure the content validity. Questionnaire was also pretested on ten randomly selected participants and after the consensus that all questions were clear and straightforward, all participants were invited to fill them in.

*Statistical Analysis*

Statistical analysis was performed using statistical package for the social sciences (SPSS) version 21 software (SPSS Inc., Chicago, IL, USA). For the purpose of analysis, each correct answer was given score “one” and wrong answer was given a score “zero” in the items included in the questionnaire. Overall, group and individual scores were based on the number of correct answers to the questions. Chi-square test was used to test the significant difference in proportions and percentages between the groups. Means were compared using t-test. P < 0.05 was considered to be statistically significant. Karl Pearson’s coefficient correlation test was used to find the correlation between KAP among the groups. 

## Results

A total of three hundred and thirty-two medical and dental undergraduate students, medical and dental GPs and specialists participated in the current study. Of the 450 HCPs approached, 332 filled the questionnaire with a response rate of 73.77%. Table 1 depicts the demographic characteristics of the participants. The result shows 75% [n=249] female participants as compared to 25% [n=83] male with a ratio of 3:1. The age of the participants ranged from 20 years to more than 50 years with majority between 20 to 30 years. 

**Figure 1:**
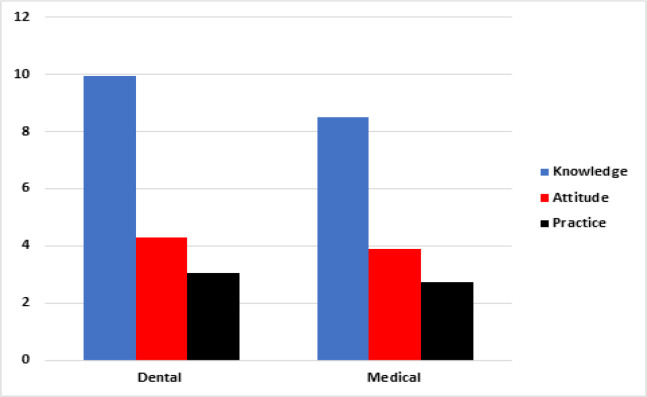
Comparison of Medical and Dental Participants with Respect to Knowledge, Attitude and Practice Scores

The results of our study demonstrated that majority of participants associated with dentistry were aware of OSCC based on the data collected and analyzed from Knowledge questions (Dental students: 87%, Dental GPs: 97%, Dental Specialists: 96%) as compared to the participants from medicine (Medical Students: 58%, Medical GPs: 43%, Medical Specialists 93%). Figure 1 and Table 2 and Table 3 shows the analysis of mean knowledge, attitude and practice index scores among dentistry and medical participants.

In order to test the knowledge regarding OSCC, participants were asked about etiological /risk factors, distribution and diagnosis. It was seen that 84.6% - 91.9% of dental participants were aware of various risk factors related to OSCC compared to 56.6% to 81.3% medical participants. Overall, only 10.5% to 21.4% of medical and dental participants agreed that alcohol and tobacco are the only responsible factors for development of OSCC. Only 37.7% of medical students were aware of symptoms related to OSCC as compared to 76.3% of dental students, whereas 57.1% of medical GPs and 93.8% of medical specialists as compared to 86.9% of dental GPs and 93.5% of dental specialists knew the symptoms of OSCC. As for the question regarding the most common site for OSCC, almost 67.3% to 79% of dental participants and 28.6% to 62.5% of medical participants agreed for the tongue to be the most prevalent site. It was noted that both medical and dental participants (31.3% to 59.3%) were of the opinion that OSCC cannot be diagnosed at early stage. It was observed that most of the respondents (Dental students: 78.8%, dental GPs: 89.5%, dental specialists: 88.7%, medical specialists: 93.8%, medical GPs: 85.7%, Medical Student:84.9%) agreed that risk of OSCC increases with age. It has been found that 

**Figure 2:**
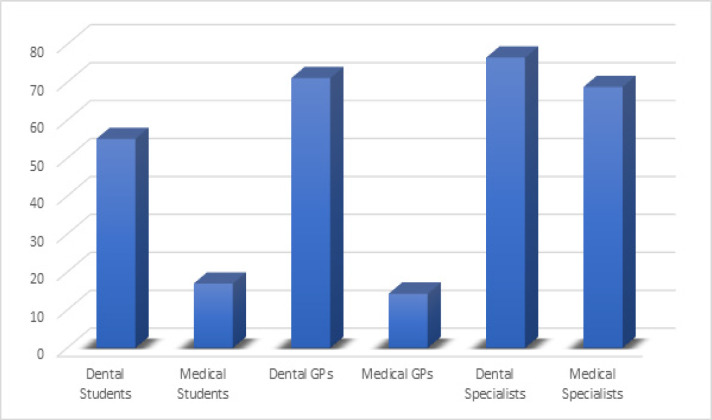
Knowledge of Health Care Professionals Regarding Investigative Procedures Done for Early Detection of OSC

71.4% to 80.4% participants from dentistry agreed that OSCC is a preventable disease compared to 57.1% to 81.9% of medical related participants. The fact that hard-painless fixed lymph node in head and neck region indicates metastasis of OSCC was identified by more than 86% of medical and dental participants. Almost 100 % of participants agreed that if they are able to detect OSCC in initial stages, it will improve the prognosis and five-year survival rate in oral cancer patients, but surprisingly many of the participants were not aware of initial investigations done for its diagnosis (Figure 2). 

Distribution of participants’ responses with answer “Yes” to questions regarding OSCC attitude and practices are demonstrated in Table 4 and Table 5. It has been found that majority of dental and medical participants agreed that regularly annual OSCC examinations should be provided specially to those who are of 40 years or above in order to improve early detection and prognosis. It was revealed that only 28.9% to 41.5% of very few HCPs are adequately trained to provide alcohol/smoking cessation education. It was noted that only 119/332 (35.8%) of respondents stated that they have information/ brochures on prevention of oral cancer in their practice area.

**Table 1:** Demographic Data of the Participants

Characteristics	Frequency (%)
Age groups	
20 – 30 y	251 (75.6 %)
31 – 40 y	55 (16.6 %)
41 – 50 y	19 (5.7 %)
More than 50 y	7 (2.1 %)
Gender	
Male	83 (25.0 %)
Female	249 (75.0 %)
Education levels	
Dental (GP)	38 (11.4 %)
Dental (Specialist)	62 (18.7 %)
Dental student	156 (47.0 %)
Medical (GP)	7 (2.1 %)
Medical (Specialist)	16 (4.8 %)
Medical student	53 (16.0 %)
Classifications	
Dental	256 (77.1 %)
Medical	76 (22.9 %)
For students (n = 209)	209 (63.1 %)-
First	36 (12.0 %)
Second	35 (10.5 %)
Third	50 (16.0 %)
Fourth	50 (15.1 %)
Fifth	38 (11.4 %)

**Table 2:** Analysis of Mean Knowledge, Attitude and Practice Index Scores among Participants from Sub-Groups of Dentistry and Medicine

Parameters		N	Mean	Std. Deviation	95% Confidence Interval for Mean		
					Lower Bound	Upper Bound	P-value
Knowledge	Dental	256	9.961	2.178	9.693	10.229	
	Medicine	76	8.487	2.341	7.952	9.022	< 0.0001
Attitude	Dental	256	4.281	1.277	4.124	4.438	
	Medicine	76	3.908	1.425	3.582	4.234	0.03
Practice	Dental	256	3.031	1.377	2.862	3.201	
	Medicine	76	2.711	1.412	2.388	3.033	0.077

**Table 3:** Mean Knowledge, Attitude and Practice Index scores among participants in each group

Parameters		N	Mean	Std. Deviation	Min	Max	P-value
Knowledge	Dental (Specialist)	62	10.629	2.313	4	15	< 0.0001
	Dental (GP)	38	10.079	1.239	7	12	
	Dental student	156	9.667	2.250	0	15	
	Medical (Specialist)	16	9.688	1.580	6	12	
	Medical (GP)	7	8.143	3.716	5	15	
	Medical student	53	8.170	2.242	4	13	
	Total	332	9.624	2.298	0	15	
Attitude	Dental (Specialist)	62	4.645	1.307	2	7	
	Dental (GP)	38	4.342	1.097	2	7	
	Dental student	156	4.122	1.282	0	7	
	Medical (Specialist)	16	4.625	1.204	2	6	< 0.0001
	Medical (GP)	7	5.000	1.155	4	7	
	Medical student	53	3.547	1.381	0	7	
	Total	332	4.196	1.319	0	7	
Practice	Dental (Specialist)	62	2.887	1.320	0	5	
	Dental (GP)	38	2.632	1.584	0	5	
	Dental student	156	3.186	1.329	0	5	
	Medical (Specialist)	16	3.000	1.366	1	5	0.075
	Medical (GP)	7	2.857	1.345	1	5	
	Medical student	53	2.604	1.446	0	5	
	Total	332	2.958	1.390	0	5	

**Table 4:** Responses “Yes” to Attitude Questions on OSCC

Health care professionals	Dental (Specialist) (n=62)	Dental (GP) (n =38)	Dental student (n =156)	Medical (Specialist) (n = 16)	Medical (GP) (n = 7)	Medical student (n = 53)
Do you feel that you are adequately trained to provide alcohol/smoking cessation education?	25 (40.30%)	11 (28.90%)	53 (34.00%)	7 (43.80%)	4 (57.10%)	22 (41.50%)
Are you adequately trained to perform patient‚ lymph node palpation?	47 (75.80%)	26 (68.40%)	105 (67.30%)	14 (87.50%)	6 (85.70%)	34 (64.20%)
Would you like more information or training on oral cancer?	59 (95.20%)	36 (94.70%)	141 (90.40%)	15 (93.80%)	7 (100%)	43 (81.10%)
Do you feel that you have sufficient knowledge of prevention and detection of oral cancer?	28 (45.20%)	18 (47.40%)	57 (36.50%)	8 (50%)	2 (28.60%)	5 (9.40%)
Is it a waste of time to educate the patients to quit their habits? (as they always decline to follow)	16 (25.80%)	4 (10.50%)	27 (17.30%)	1 (6.30%)	2 (28.60%)	2 (3.80%)
Should patients with suspected oral cancerous lesions be referred to a specialist?	62 (100%)	38 (100%)	138 (88.50%)	16 (100%)	7 (100%)	49 (92.50%)

**Table 5:** Responses to Practice Questions on OSCC

Health care professionals	Dental (Specialist) (n=62)	Dental (GP) (n =38)	Dental student (n =156)	Medical (Specialist) (n = 16)	Medical (GP) (n = 7)	Medical student (n = 53)
Do you examine patients and oral mucosa routinely?	46 (74.20%)	26 (68.40%)	109 (69.90%)	7 (43.80%)	2 (28.60%)	14 (26.40%)
If “No” to the above , do you screen the oral mucosa if the patients are in high categories?	28 (77.80%)	12 (60.00%)	54 (63.50%)	6 (50.00%)	4 (66.70%)	21 (56.80%)
Do you record tobacco and alcohol use in personal history?	45 (72.60%)	29 (76.30%)	132 (84.60%)	16 (100%)	6 (85.70%)	46 (86.80%)
Do you practice complete oral cavity examination besides palpating lymph nodes routinely?	35 (56.50%)	18 (47.40%)	123 (78.80%)	9 (56.30%)	3 (42.90%)	23 (43.40%)
Do you take biopsy in patients with suspicious lesions?	25 (40.30%)	15 (39.50%)	79 (50.60%)	10 (62.50%)	5 (71.40%)	34 (64.20%)

## Discussion

OSCC is considered as one of most prevalent cancers world-wide. It is emerging as a major health problem. It has been observed that now a days more women and youngsters are being affected by this malignancy (Gupta et al., 2016). In Saudi Arabia also, oral cancer is very common and it is estimated that OSCC accounts for almost 26 % amongst all the head and neck cancers which are detected annually in KSA and majority of these cases are diagnosed in advanced stages (Basha et al., 2019; Jaber et al., 2012). It has been reported that early diagnosis of OSCC increases the likelihood of cure thus improving the survival rate and reducing the associated morbidities (Sciubba and Larian, 2018). HCPs need to have a thorough knowledge regarding etiological/risk factors of OSCC along with familiarity with its early signs and symptoms so that they can identify and refer or treat the patients in initial stages. The present study was conducted with the aim that understanding the knowledge, attitude and practices is of utmost importance for both medical and dental professionals in prevention and early detection of OSCC. In our study, three hundred and thirty-two HCPs were given a questionnaire assessing awareness of OSCC.

In the present study, it was noted that the majority of participants from both medical and dental backgrounds were aware of OSCC but the knowledge scores were significantly higher in dental professionals as compared to medical professionals. These findings are in agreement with study conducted in Jordan in which knowledge of oral cancer was assessed among medical and dental professionals in King Hussein Cancer Center (KHCC) in Amman, Jordan, revealing an adequate level of knowledge of oral cancer with substantial differences between the dental and medical professionals (Alami et al., 2013).

The occurrence of OSCC has been reported at various sites such as lips, tongue, salivary glands, pharynx and larynx. It has been observed that patients with numbness, pain/tenderness, ulcers, swellings in the mouth as well as lips and patients having difficulty during swallowing or speaking, seek help from their general physician first. Therefore, both medical and dental practitioners and students must have sufficient and up to date knowledge regarding OSCC to ensure early diagnosis which is a key to successful treatment and better prognosis (Carter and Ogden, 2007). 

Our study found that majority of participants were able to identify that tobacco use is one of the main risk factors for OSCC and combination of smoking and alcohol is more harmful which is consistent with available literature also (Alami et al., 2013) in which it is identified that majority of HCPs are aware of effects of tobacco on OSCC. In the present study, majority of medical/dental students and practitioners reported that they always record tobacco use in patient’s history. However, surprisingly most of respondents (Medical participants: 47.46%; dental participants 34.4%) were of the opinion that they were not adequately trained to provide alcohol/smoking cessation advice during their undergraduate education. Recent studies have specified that one third of new cases of OSCC can be circumvented by eradicating or reducing exposure to known risk factors such as tobacco and alcohol (Islami et al., 2018; Wilson et al., 2018). Proper maintenance of records and counselling for quitting the habit of smoking is very important as frequent use of smokeless tobacco (Shammah and Quat) is considered as a main reason for high prevalence of oral cancer in Saudia (Basha et al., 2019).

In our study, 71.2% to 85% of participants believed that OSCC is a preventable disease and 86.7% to 100% of participants recognized the fact that early detection can improve 5-year survival rate. In spite of the fact that the oral cavity is accessible for visual and clinical examination, OSCC are usually diagnosed at advanced stages (Coelho, 2012). In our study, it was identified that 28.9% to 51.7% of medical and dental professionals feel that they have sufficient knowledge concerning prevention and detection of this tumor and reported the need for more information or training on OSCC facts which was consistent with a previous study (Carter and Ogden, 2007) . It is worth mentioning that only 26.4% to 43.8% of medical and 69.9% to 74.2% of dental professionals were in habit of examining oral mucosa. For high risk patients, almost 56.8% to 77.8% of medical and dental professionals screen the mucosa. The findings of our study are in agreement with Janaswer study (Jnaneswar et al., 2017). In addition, it was observed that 88.5% to 100% of participants referred patients with suspected malignant lesion to a specialist. The results of our study are different from Ahmad and Naidoo study in which only 52.2% of referrals were reported (Ahmed and Naidoo, 2019). 

The findings of the present study suggested that both dental and medical participants were well aware about OSCC and had a good knowledge about associated risk factors. However, participants of the present study indicated their lack of training as the main barrier for conducting a comprehensive oral examination for early cancer detection and diagnosis. Interestingly, the vast majority of the participants expressed their interest to have further educational and training sessions on various aspects of OSCC.

In conclusion, both medical and dental professionals must have adequate knowledge and training for early and effective diagnosis of malignant and premalignant lesions observed in the oral cavity. There should be ample literature available for the patients in the healthcare centers for tips on cessation of deleterious habits and their harmful effects on health. moreover, special workshops should also be conducted for the HCPs to train them in providing guidance to the patients regarding cessation of habits which have a contributing role in the etiology of OSCC. The educational and training interventions in medical and dental curriculum are necessary to improve diagnostic skills of medical and dental professionals with respect to intra- oral lesions which may lead to improved mortality and morbidity of patients suffering from OSCC. 
